# Serial Homology and Segment Identity in the Arthropod Head

**DOI:** 10.1093/iob/obac015

**Published:** 2022-04-21

**Authors:** Oren Lev, Gregory D Edgecombe, Ariel D Chipman

**Affiliations:** The Dept. of Ecology, Evolution & Behavior, The Silberman Institute of Life Sciences, The Hebrew University of Jerusalem, Jerusalem, Israel; Department of Earth Sciences, The Natural History Museum, London, UK; The Dept. of Ecology, Evolution & Behavior, The Silberman Institute of Life Sciences, The Hebrew University of Jerusalem, Jerusalem, Israel

## Abstract

The anterior-most unit of the crown-group arthropod body plan includes three segments, the pre-gnathal segments, that contain three neuromeres that together comprise the brain. Recent work on the development of this anterior region has shown that its three units exhibit many developmental differences to the more posterior segments, to the extent that they should not be considered serial homologs. Building on this revised understanding of the development of the pre-gnathal segments, we suggest a novel scenario for arthropod head evolution. We posit an expansion of an ancestral single-segmented head at the transition from Radiodonta to Deuteropoda in the arthropod stem group. The expanded head subdivided into three segmental units, each maintaining some of the structures of the ancestral head. This scenario is consistent with what we know of head evolution from the fossil record and helps reconcile some of the debates about early arthropod evolution.

## Abstract (he)

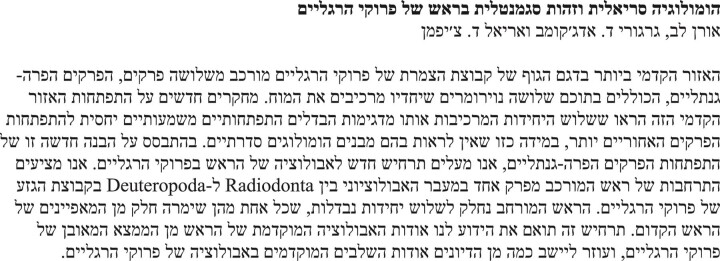

## Abstract (ar)

Arabic

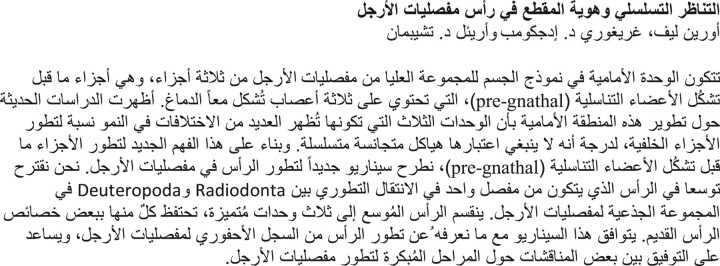

## Abstract (fa)

Persian

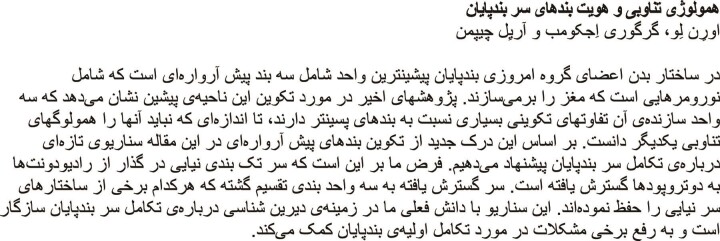

## Russian

Серийная Гомология и Принадлежность Сегментов Головы у Членистоногих

Орэн Лев, Грегори Д. Эджкомб и Ариэль Д. Чипман

Самый передний элемент плана строения тела членистоногих коронной группы включает три сегмента, которые являются предшественниками гнатальных сегментов, содержащие три нейромера, что в совокупности составляют мозг. Недавнее исследование развития указанного переднего участка показало, что три его элемента демонстрируют настолько много различий в развитии по сравнению с более задними сегментами, что их не следует считать серийными гомологами. Основываясь на этой пересмотренной концепции развития предшественников гнатальных сегментов, нами предложен новый вариант эволюции головы членистоногих. Мы предполагаем, что увеличение предковой односегментной головы произошло при переходе от Radiodonta к Deuteropoda в стволовой группе членистоногих. Увеличенная голова разделилась на три сегментарных элемента, каждый из которых сохранил некоторые структуры предковой головы. Этот вариант соответствует тому, что нам известно об эволюции головы из описаний окаменелостей, и помогает решить некоторые споры о ранней эволюции членистоногих.

## Ukrainian

Серійна Гомологія та Приналежність Сегментів Голови у Членистоногих

Орен Лев, Грегорі Д. Еджкомб та Аріель Д. Чіпман

Найпередніший елемент плану будови тіла членистоногих коронної групи включає три сегменти, які є попередниками гнатальних сегментів, що містять три нейромери, які в сукупності складають мозок. Нещодавнє дослідження розвитку зазначеної передньої ділянки показало, що три його елементи демонструють настільки багато відмінностей у розвитку у порівнянні з більш задніми сегментами, що їх не варто вважати серійними гомологами. На основі цієї переглянутої концепції розвитку попередників гнатальних сегментів, ми пропонуємо новий варіант еволюції голови членистоногих. Ми вважаємо, що збільшення предкової односегментної голови відбулося при переході від Radiodonta до Deuteropoda у стовбурній групі членистоногих. Збільшена голова розділилася на три сегментарні елементи, кожен із яких зберіг деякі структури предкової голови. Цей варіант узгоджується з тим, що нам відомо про еволюцію голови з описів скам'янілостей, і допомагає вирішити деякі суперечки про ранню еволюцію членистоногих.

## French

Homologie en série et identité des segments dans la tête de l'arthropodes

Oren Lev, Gregory D. Edgecombe and Ariel D. Chipman

L'unité la plus antérieure du plan corporel des arthropodes du groupe couronne comprend trois segments, les segments pré-gnathaux, qui contiennent trois neuromères qui, ensemble, constituent le cerveau. Des études récentes sur le développement de cette région antérieure ont montré que ses trois unités présentent de nombreuses différences de développement avec les segments plus postérieurs, au point qu'elles ne doivent pas être considérées comme des homologues sérielles. Basés sur cette révision de notre compréhension du développement des segments pré-gnathaux, nous proposons un nouveau scénario pour l'évolution de la tête des arthropodes. Nous postulons une expansion d'une tête ancestrale mono-segmentaire lors de la transition de Radiodonta à Deuteropoda dans le groupe souche des arthropodes. La tête élargie était divisée en trois unités segmentaires, chacune conservant certaines des structures de la tête ancestrale. Ce scénario est cohérent avec ce que nous savons de l'évolution de la tête à partir des archives fossiles et aide à concilier certains débats sur les stades premiers de l'évolution des arthropodes.

## Spanish

Homología serial e identidad de los segmentos en la cabeza del artrópodos

La parte más anterior del plano corporal del grupo corona de los artrópodos incluye tres segmentos, los llamados segmentos pregnatales, que a su vez contienen tres neurómeros que constituyen el cerebro en su conjunto. Estudios recientes sobre el desarrollo de esta región anterior han demostrado que sus tres unidades muestran muchas diferencias de desarrollo con respecto a los segmentos posteriores, por lo que no deberían considerarse homólogos seriales. Teniendo en cuenta el desarrollo de los segmentos pregnatales, sugerimos un escenario alternativo para la evolución de la cabeza de los artrópodos. Proponemos la expansión de una cabeza ancestral con solo un segmento en la transición evolutiva de Radiodonta a Deuteropoda en el grupo troncal de los artrópodos. La cabeza expandida se subdividió en tres unidades segmentales, cada una de ellas manteniendo algunas de las estructuras de la cabeza ancestral. Este escenario es consistente con nuestro conocimiento de le evolución cefálica en el registro fósil y facilita la reconciliación de algunos de los debates sobre las etapas tempranas en la evolución de los artrópodos.

## Italian

Omologia seriale e identità degli segmenti nella testa degli artropodi

La parte più anteriore dell'asse del corpo del gruppo crown degli artropodi comprende tre segmenti, i segmenti pre-mandibulari, che a loro volta contengono tre neuromeri che costituiscono l'encefalo nel suo insieme. Studi recenti sullo sviluppo di questa regione anteriore hanno dimostrato che le tre unità che la compongono mostrano molte differenze rispetto ai segmenti più posteriori, al punto che non dovrebbero essere considerate omologhi seriali dei segmenti più posteriori. Sulla base di questa riveduta interpretazione dello sviluppo dei segmenti pre-mandibulari, suggeriamo uno scenario alternativo per l'evoluzione del capo degli artropodi, ovvero l'espansione di una regione cefalica ancestrale con un solo segmento nel passaggio evolutivo da Radiodonta a Deuteropoda, nel gruppo stem degli artropodi. Il capo espanso si sarebbe diviso in tre unità segmentali, ciascuna delle quali conserva alcune delle strutture del capo ancestrale. Questo scenario è coerente con le conoscenze sull'evoluzione del capo nella documentazione fossile e aiuta a conciliare opinioni diverse nel dibattito sulle prime fasi dell'evoluzione degli artropodi.

## Background

Arthropods have been the dominant animals on Earth since the early Cambrian. Today, they are the most species-rich phylum, and in almost all invertebrate fossil assemblages with exceptional preservation they are the most prolific group represented. In the Cambrian fossil record, we find a mix of species descended from the most recent common ancestor of extant arthropods—so called crown-group arthropods—together with species that branched off before the appearance of this ancestor—the stem-group (Edgecombe 2020). The earliest assemblages contain a mix of coeval stem- and crown-group arthropods and present a series of character states that can inform about the evolution of key arthropod features (Giribet and Edgecombe 2019). Fossils representing the stem-group do not display all the characters that define extant arthropods. Notably, they display diverse head structures and head segmental organizations that differ substantially from those found today (Ortega-Hernández et al. 2017).

The debate about the specific homologies of the head-related structures in fossil arthropods and their relatives, and indeed, what constitutes the “head,” remains one of the great unresolved questions in arthropod evolution. The question of homology of segmental structures in extant arthropod heads has been resolved in the past 20 years (Scholtz 2015; Ortega-Hernández et al. 2017) through a combination of embryonic gene expression (Damen et al. 1998; Telford and Thomas 1998; Posnien et al. 2010; Janssen et al. 2011) and neuroanatomy (Mittmann and Scholtz 2003; Loesel et al. 2013). It is now well accepted that extant arthropod heads include a conserved anterior region composed of three segments and that the appendages of these three segments can be homologized across extant arthropods. Each of these anterior segments contains a large dorsal ganglion, and these three ganglia together comprise the arthropod brain. The segments are usually named after the ganglia they contain; the protocerebral, deutocerebral, and tritocerebral segments are the first, second, and third anteriormost segments, respectively. Alternatively, they are named after the appendages they carry, which differ between arthropod classes. In mandibulates, the deutocerebral segment carries the first pair of antennae and the tritocerebral segment carries the second pair of antennae, or in the case of hexapods and myriapods, lacks an appendage. In chelicerates, the deutocerebral appendage pair is the chelicerae (chelifores in pycnogonids) and the tritocerebral appendages are the pedipalps. In all arthropods, the protocerebral segment carries the eyes either in the form of eye spots, simple eyes, or compound eyes, the latter either stalked or sessile. Although eyestalks reveal anatomical and physiological correspondences with appendages (reviewed by Strausfeld et al. (2016)), eyes are not usually ascribed an appendicular identity, being generated by a distinct gene regulatory network different from appendages (Friedrich 2003). There has, however, been little work on the developmental genetics of stalked eyes in extant arthropods, so an appendicular origin cannot be refuted. The protocerebrum also bears the labrum, a structure that expresses appendage-related genes during development (Browne et al. 2005; Kimm and Prpic 2006; Posnien et al. 2009; Jockusch 2017). Because in insects, the three segments lie anterior to the mouthparts, they are referred to more generally as the pre-gnathal segments (PGS). We use this term here for consistency, acknowledging that these segments are not actually anterior to gnathal elements in all arthropods. We refer to all other segments as “post-gnathal” or “trunk” segments to distinguish them from the three segments of the anterior region.

**Fig. 1:**
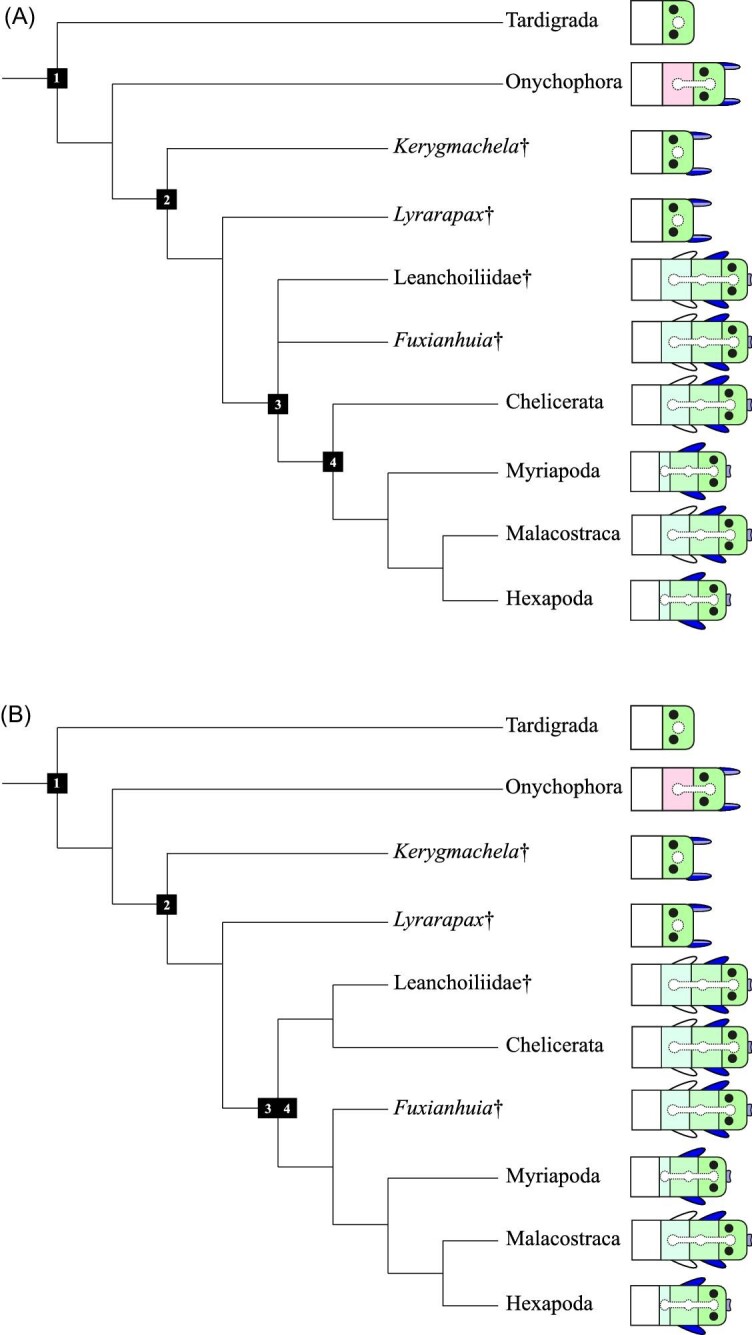
The change in head structure mapped on alternative phylogenetic trees of Panarthropoda. (1) Panarthropoda. (2) Total group Arthropoda (stem + crown). (3) Deuteropoda. (4) Crown group Arthropoda. Each taxon is represented by a scheme of the head and the first trunk segment (marked in white). In lower-stem taxa the head is composed of the protocerebrum only (including the prosocerebrum), marked in green. Evidence for eyes in *Kerymachela* (Park et al. 2018) is equivocal (Lan et al. 2021). In Deuteropoda (Node 3) the head is shown as composed of three segments, marked in different shades of green to indicate their shared ancestry; other interpretations of fossilized neuroanatomy delay the change from a protocerebral brain to a tripartite brain to node 4 (Budd 2021). The dorsal ganglion/ganglia are marked with a dotted outline within the head segments. The anterior raptorial appendage is shown on the anterior, prosocerebral domain in lower-stem taxa in two shades of blue. In Deuteropoda the labrum is shown as an anterior medial structure in light blue and the deutocerebral appendage is shown in dark blue, indicating their putative shared derivation from the original anterior raptorial appendage. Eyes are represented as black circles in the protocerebral segment. The third (tritocerebral) segment is shown to be reduced in Myriapoda and Hexapoda, where it becomes the appendageless intercalary segment. The onychophoran second segment is shown in pink, to indicate that it is probably not homologous to the deutocerebral segment in Deuteropoda. The relationships of extant taxa are based on phylogenomic data. (A) Fuxianhuiids and leanchoiliids interpreted as stem-group arthropods. The relative positions of *Fuxianhuia* and Leanchoiliidae alternate in published trees adopting this framework (Budd 2021, fig. 9B versus Legg et al. 2013), so they are shown as a trichotomy with crown-group Arthropoda. (B) Fuxianhuiids and leanchoiliids interpreted as crown-group arthropods (topology from Zeng et al. 2020, Extended Data, Fig. 9a).

In contrast with the resolution in extant arthropods, the homology of anterior segments and their appendages in fossil arthropods remains in flux, with some aspects resolved and others hotly contested. It is reasonably well-established that several fossil taxa, relatively distantly related to crown-group arthropods, had a head composed of a single segment. Evidence for this comes from exceptionally preserved fossils of the stem-group arthropods *Kerygmachela* (Park et al. 2018) and *Lyrarapax* (Cong et al. 2014) that preserve remains of neural tissue suggesting that the brain of these species was composed of a single neuromere. This single neuromere has been attributed to the protocerebrum, as in the brain of tardigrades (Smith et al. 2017; Gross et al. 2021). Since phylogenomic data support a sister group relationship between tardigrades and the other two panarthropod phyla, Onychophora and Arthropoda (Campbell et al. 2011) (Fig. 1), these data together suggest a single-segment head to be primitive for Panarthropoda as a whole (Ortega-Hernández et al. 2017; Park et al. 2018). Additional evidence for an ancestral single-segment head is provided by the presence of a single pair of head appendages, large raptorial appendages, in a range of stem-group taxa. In the rare fossils with preserved neural tissue, this appendage pair either houses a nerve emanating from the anteriormost part of a unipartite brain (Park et al. 2018) or, when the frontal appendage is arthropodized, is served by a frontal ganglion (Cong et al. 2014).

The situation is complicated by evidence from extant arthropods that suggests the protocerebrum is composed of two domains, the prosocerebrum and the archicerebrum (Urbach and Technau 2003). The prosocerebrum includes several main processing centers such as the central body and neurosecretory cells, and innervates the labrum (Urbach and Technau 2003; Lan et al. 2021). The archicerebrum innervates the compound eyes. The prosocerebrum and its associated structures are generally considered to represent an asegmental anterior region (Posnien et al. 2009; Steinmetz et al. 2010), which may be homologous to an anterior region that expresses the homeobox gene *Six-3/optix* in all bilaterians (Steinmetz et al. 2010).

The frontal pair of appendages in those fossil taxa with a single-segment head is attributed to the prosocerebrum (Lan et al. 2021), and they are thus interpreted as being homologous to the labrum (Ortega-Hernández et al. 2017; Budd 2021). Under this interpretation, the compound eyes of *Lyrarapax* and its relatives, the radiodonts such as *Anomalocaris*, are innervated by the archicerebrum, like the compound eyes of extant arthropods. The radiodont head shows evidence for being bipartite, the genus *Amplectobelua* having an oval head shield likely aligned with the frontal appendages and a pair of plates called P-elements associated with the eyes (Cong et al. 2017) but collectively these can be ascribed to the two domains of the protocerebrum.

Some Cambrian taxa have been argued to show a head composed of three segments, corresponding to the PGS of extant arthropods. Notably, exceptionally preserved fossils of the Cambrian arthropod *Fuxianhuia* are consistent with a tri-partite brain (Ma et al. 2012), each neuromere of which innervates one anterior head segment. Leanchoiliid “great appendage arthropods” likewise have been attributed proto-, deuto-, and trito-cerebral segmental input to the brain, with the deutocerebrum innervating the anteriormost appendage; this tripartite brain is preceded by a prosocerebrum that sends nerves to ganglia that innervate the labrum (Lan et al. 2021). However, the neuroanatomical interpretations of these fossils (both fuxianhuiids and leanchoiliids) having tripartite brains was disputed by Budd (2021), who proposed alternative schemes in which they instead have single-segment (protocerebral) brains as in more stemward arthropods like *Kerygmachela* and Radiodonta.

Many Cambrian arthropods with tri-partite brains display anterior raptorial appendages that are often similar in structure to those found in the taxa with a single-segment head (Chen et al. 2004; Haug et al. 2012; Aria and Caron 2015; Zeng et al. 2020). The raptorial appendage is undoubtedly found on the anterior-most, single head segment in taxa that are low on the arthropod stem. However, there are two opposing views as to the segmental identity of the raptorial appendage in the more crownward fossils with a tri-partite head such as leanchoiliids. Some authors argue that the raptorial appendage belongs to the deutocerebral segment and is therefore not homologous to that found in the lower-stem (Chipman 2015; Ortega-Hernández et al. 2017). Others argue that the great appendages of different taxa are similar enough and have the same topological relationships to other parts of the head (such as the eyes and an anterior sclerite) to argue for homology and therefore must all be located on the same segment (Haug et al. 2012; Aria and Caron 2015; Aria et al. 2020).

In a framework in which fuxianhuiids, leanchoiliids, and other fossil taxa with (at least) a three-segmented head are assigned to the arthropod stem group (Fig. 1A), they and crown-group arthropods are collectively named Deuteropoda, for the presence of deutocerebral appendages (Ortega-Hernández 2016). However, the status of fuxianhuiids and leanchoiliids as stem-group arthropods has been challenged by phylogenetic analyses that instead place them inside the arthropod crown group, as stem-group Mandibulata and as stem-group Chelicerata, respectively (Zeng et al. 2020; Aria et al. 2021); under this phylogeny, Deuteropoda is equivalent to crown-group Arthropoda (Fig. 1B). We stress that the hypothesis for the evolution of the PGS we develop below is robust to the discordance between these two alternative phylogenetic schemes when both agree that the contested fossils have tripartite brains.

In essence, the difficulty with understanding the early evolution of the arthropod head hinges on the transition from the lower-stem arthropods to Deuteropoda. Over a single node on the currently best supported phylogenetic trees, a large number of character state transformations occur (Fig. 1). The single-segment head is transformed into a three-segment head, with a one-neuromere brain turning into a three-neuromere brain. The anterior raptorial appendage is reduced to a labrum, while in parallel, a new appendage appears on the deutocerebral segment. The trunk appendages undergo arthropodization, possibly through recruitment of the limb patterning gene regulatory network from the anterior raptorial appendage to the trunk appendage (Chipman and Edgecombe 2019). This is a dramatic series of concerted changes that is difficult to reconcile with our understanding of the evolution of morphology (Chipman 2015). The fact that there are currently no known fossils that exhibit an intermediate stage of this transition suggests that it happened fairly rapidly, and that these character state transformations may arise from the same process.

## Development of the PGS

The understanding that the three PGS in insects are developmentally different from the gnathal segments of the head is not new (Gallitano-Mendel and Finkelstein 1997). Development of the PGS is not regulated by pair-rule genes (Rogers et al. 2002; Choe and Brown 2007; Posnien and Bucher 2010), and Hox genes are not expressed in the two anterior pre-gnathal segments. The PGS form earlier than or at the same time as post-gnathal segments (Schönauer et al. 2016; Stahi and Chipman 2016; Hunnekuhl and Akam 2017), their structure during the embryonic germband stage is very different from the structure of all other segments, and they are arranged in distinctive structures known as the head lobes. Previous work suggested that these differences are consequences of the divergent structure of the anterior head segments (Gallitano-Mendel and Finkelstein 1997), implicitly suggesting that segmental patterning of the PGS underwent changes relative to the original state, following their incorporation into the six-segment head of extant insects.

Recently, Lev and Chipman (2021), reviewing data from the literature and introducing new data for the milkweed bug *Oncopeltus fasciatus*, showed that the gene regulatory network underlying the development of the PGS is fundamentally different from that of all other segments. One of the findings from that study is stripe splitting in the expression of the segment polarity gene *hedgehog* during PGS formation in *Oncopeltus*. Expression of *hedgehog* begins as a single stripe that splits into two domains that act as borders within the PGS. This pattern of *hedgehog* expression pattern in the developing PGS can also be seen in chelicerates, myriapods, and other insects (Miyawaki et al. 2004; Farzana and Brown 2008; Kanayama et al. 2011; Janssen 2012; Hunnekuhl and Akam 2017; Barnett and Thomas 2018) and can thus be inferred for the last common ancestor of crown-group arthropods. In chelicerate and myriapod head development, *hedgehog* stripe splitting results in either two or three stripes, while in all studied insect species, it ends with two stripes. This expression pattern is unlike *hedgehog*’s conserved segmental expression pattern seen in the post-gnathal segments of arthropods. In a series of parental RNAi experiments, Lev & Chipman (2021) showed different interaction between the segment polarity genes in the PGS compared with post-gnathal segments. They also showed that when *hedgehog* is knocked down, the outcome is severely reduced anterior appendages and structures such as the labrum and antenna in affected embryos, with no effect on post-gnathal segmental appendages. This functional manipulation of *hedgehog* expression has not been done in other arthropod species, at least not during the development of the pre-gnathal segments. However, *hedgehog* expression patterns in the relevant time frame of development across arthropods are nearly identical, so deducing a similar function is valid, pending further investigation of additional species.

Lastly, several studies noted that *engrailed*, a segment polarity gene crucial to segmentation, is expressed later than *hedgehog* in the PGS and after the segments are morphologically evident (Brown et al. 1994; Gallitano-Mendel and Finkelstein 1997; Janssen 2012; Stahi and Chipman 2016). This makes it unlikely that *engrailed* plays an important role in early PGS formation, as it does in the other segments.

Based on this body of evidence that includes difference in developmental timing, morphology and genetic interactions, Lev & Chipman (2021) suggested that the PGS do not share a developmental gene regulatory network with all the other segments and therefore should not be seen as serially homologous, in the sense of not being patterned by the same network (Wagner 2014; Tomoyasu et al. 2017). This lack of serial homology indicates a separate evolutionary trajectory, and probably a different evolutionary source for the pre-gnathal segments.

## The development of segmental identity

The process of segment generation is a tiered process, consisting of several inter-dependent developmental steps. While there is a significant amount of diversity in some of the steps, the general principles are conserved across arthropods (Peel et al. 2005). The first stage in the process is the generation of a repeated pre-pattern. This is the most variable stage, with differences in the dynamics of setting up the pre-pattern and in the precise identity of the genes involved (Peel et al. 2005; Clark et al. 2019). These differences are detected both between species and even within the embryo of a single species, where different segments can be pre-patterned through different processes. Examples include sequential segmentation vs. simultaneous segmentation between gnathothoracic and abdominal segments in some insects (Stahi and Chipman 2016; Auman et al. 2017) and between prosomal and opisthosomal segments in spiders (Hemmi et al. 2018). Comparative work on arthropod segmentation shows that even very similar segments can be pre-patterned through different processes.

The next tier is the determination of segment boundaries. This is the most conserved stage of the process (Damen 2002; Janssen et al. 2004; Peel et al. 2005; Janssen 2012; Auman and Chipman 2017; Chipman 2020). A group of genes known as segment-polarity genes (which includes the aforementioned *hedgehog*) interact in a conserved regulatory network to generate a stable molecular boundary between adjacent segments (von Dassow et al. 2000). This process is common to all segments in all arthropods studied to date *with the exception of the pre-gnathal segments*, for which the interactions and dynamics of the segment polarity genes are different (Lev and Chipman 2021). We identify the segment polarity network as the character identity network (ChIN—*sensu*Wagner (2007)) of the arthropod segment (DiFrisco and Wagner 2022). The different network in the PGS led Lev and Chipman (2021) to suggest that they are not serial homologs of the trunk segments.

The third tier is the conferring of segmental identity—the specific structure of the segments and of segmental appendages. This is mediated largely by the Hox genes, although there is probably some input from the genes of the pre-pattern stage (Auman and Chipman 2017). While Hox genes are conserved in arthropods, and indeed across all Bilateria, the boundaries between their expression domains are variable between the different higher taxa within arthropods (Hughes and Kaufman 2002). The extent of Hox expression domains is linked to differences in segmental identity (Akam 1998a; 1998b).

Based on this developmental understanding of how segments form and assume their identity, we suggest a novel scenario for arthropod head evolution that can defuse many of the inherent difficulties in our current understanding of the process.

## Implication for the evolution of the arthropod head

A segmented body with an anterior head tagma predates the most recent common ancestor of arthropods (Budd 2002; Edgecombe and Legg 2014; Chipman and Edgecombe 2019; Giribet and Edgecombe 2019). The presence of a single brain neuromere in both tardigrades (Smith et al. 2017; Gross et al. 2021) and in lower-stem arthropods (Cong et al. 2014; Park et al. 2018) suggests that this is the primitive condition for Panarthropoda. Thus, the earliest branching stem-group arthropods, exemplified by *Kerygmachela* and we presume by even more stemward lobopodian total-group arthropods that share a similar annulated, non-arthropodized frontal appendage (*Jianshanapodia, Megadictyon*, and *Siberion*; (Dzik 2011; Edgecombe 2020)) probably had a head made up of a single unit—the protocerebrum, with a single raptorial appendage pair. Most models for the evolution of the arthropod head (e.g., (Mayer et al. 2010; Ortega-Hernández et al. 2017) suggest that the transition to a three-segmented head occurred through the recruitment of two trunk segments into the head region. Implicitly, the suggestion is that the second PGS, the deutocerebral segment, is homologous with the first trunk segment of lower stem arthropods and tardigrades, and the third PGS, the tritocerebral segment, is homologous with the second trunk segment. If this scenario were true, we would expect at least the two “new” head segments to be serially homologous to the trunk segments, that is, to develop using the same character identity network—the canonical segment-polarity gene regulatory network. The embryological data introduced in the previous sections are not consistent with such a scenario.

We suggest that the transition between a single-segmented head in lower-stem arthropods and a three-segmented head in Deuteropoda involved the elaboration of an ancient single head unit into three (Fig. 1). In the early history of arthropods, lobopodians like *Jianshanapodia* and *Megadictyon* depict a body made up of a series of homonomous trunk segments, with a single unit making up the head and in it (fide the more crownward *Kerygmachela*) a single neuromere functioning as a brain. This anterior unit was different to all segments by virtue of it being an apical unit, that is, a unit that is connected to other units on only one side. Evidence from fossils (Cong et al. 2014; Lan et al. 2021) is consistent with neural lineage-specific domains of the embryonic insect brain (Urbach and Technau 2003; Richter et al. 2010; Steinmetz et al. 2010) that suggest this apical structure was already differentiated into two regions—the prosocerebrum and the archicerebrum. We suggest that the original head was patterned and differentiated via a separate developmental pathway than the trunk segments, using some of the same genes that were used in the trunk segments, but with different interactions. As the brain expanded, it elaborated into a tri-partite brain by adding new neuromeres that subdivided from the existing neuromeres, concomitantly splitting the surrounding morphological structures into three distinct units and incorporating new developmental processes to pattern the new head. The three new units are not “regular” segments, but subdivisions of the ancestral head that have elaborated to take on certain segmental characteristics. The stripe splitting of *hedgehog* preserves an evolutionary remnant of this process.

It is worth reiterating that the anteriormost unit, the protocerebral segment, is in itself made up of two distinct units, so the arthropod brain might also be described as “quadripartite.” However, we prefer to maintain the conventional terminology of a tri-partite brain and anterior head, following the expression of three stripes of *hedgehog* and other segmental genes. Also worth noting is that the two-segment brain of Onychophora represents a convergent expansion of the ancestrally protocerebral brain under any scenario (Martin et al. 2022).

We can draw a heuristic analogy with molecular evolution. When a gene undergoes duplication, often each new copy takes on part of the roles originally carried out by the parent gene, a phenomenon known as sub-functionalization. We suggest that when the ancestral head split to become what is now recognized as a three-segmented head, each of the new subdivisions took up some of the structures and functions of the ancestral head. This suggestion provides a possible solution to the debate regarding the homology of the deutocerebral raptorial appendage of Deuteropoda such as leanchoiliid “great appendage” arthropods and *Kylinxia* (Zeng et al. 2020) and the frontal raptorial appendage of lower-stem arthropods such as *Kerygmachela* and radiodonts. When the ancestral single head segment split, the second of the resulting segments (the deutocerebral segment) inherited the raptorial appendage of the original single segment. The two appendages can thus be seen to be homologous, despite their different segmental position, as already alluded by Zeng et al. (2020). This distribution of characters among the three new segments might also explain the partial appendage identity of the labrum, as the new protocerebral segment maintained the appendage patterning network, without the axial elaboration of the original protocerebral appendage. The single-axis structure of the deutocerebral appendages in extant (and fossil) arthropods can also be attributed to its origin from the primitively single-axis appendage of lower stem arthropods. In contrast, all appendages derived from the post-gnathal segments primitively display a biramous structure (Boxshall 2004; Boxshall 2013). There are rare cases of deutocerebral appendages with a biramous structure (e.g., malacostracan antennules and pauropod antennae), but these are clearly derived. The biramous state of many tritocerebral appendages may be due to adoption of partial trunk identity, as we describe below.

Indeed, we can say the three PGS are not serial homologs of each other, but rather—adopting once again the terminology from gene evolution—paralogs of each other. After splitting, they continued to evolve independently, free from the constraints of a shared gene regulatory network. This is consistent with the differences in the specifics of gene expression among the different PGS (Lev and Chipman 2021). The degradation of the tritocerebral segment to a rudimentary intercalary segment in insects led to reduced and late expression of several of the segment-polarity genes in the insect intercalary segment.

Later in arthropod evolution, following the chelicerate-mandibulate split, additional segments were recruited to the three-segment head to give the six-segment head of mandibulates and the seven-segment prosoma of chelicerates. These additional segments were normal trunk segments that were integrated into the head/prosoma and changed their adult morphology but maintained their embryological similarity to the limb-bearing thoracic/opisthosomal segments. These segments continue to be patterned through the canonical segment-polarity network, despite being recruited to the head. Their former trunk identity was preserved with their expression of pair-rule genes and the canonical expression of *engrailed*, both of which are missing in PGS development.

A possible caveat to this model is the fact that in many cases, the appendage of the tritocerebral segment is indistinguishable from that of a trunk segment. This can be seen in the pedipalps of horseshoe crabs and in the biramous tritocerebral appendages of the upper-stem (Fig. 1A) or total-group chelicerate (Fig. 1B) fossils of the Leanchoiliidae. This trunk-like identity of PGS can potentially be explained by the intrusion of anterior Hox expression into the tritocerebral segment. As detailed above, segment identity is largely conferred by Hox expression, independently of the mode of segment generation, and in a separate and later developmental stage. We suggest that some aspects of the morphology of the tritocerebral segments may be controlled by the anterior Hox genes that are expressed there and are linked with the evolution of head structures in many bilaterians (Hombria et al. 2021). The distinct evolutionary history of the tritocerebral segment is still evident in the earlier stage of segment generation.

This phenomenon of a mismatch between the evolutionary history of the segment and its developmental identity is similar to that shown in the evolution of the wing during the dinosaur-bird transition. Wagner and Gauthier (1999) argued that digits 2, 3, 4 adopt morphological identities of digits 1, 2, 3, leading to the mismatch. Similarly, the tritocerebral segment adopts a trunk-like identity mediated by Hox expression.

## Conclusions

We posit that the three PGS have an evolutionary origin that is independent from post-gnathal segments and suggest they evolved through the expansion of an ancestral single-segment head. This new insight enables reinterpretation of the changes in head morphology throughout arthropod evolution, as represented in the fossil record. It also opens the door for more detailed analyses of the development of the head in extant arthropods with the aim of reconstructing the precise changes in developmental regulation that led to the evolution of the complex head we see today.

## References

[bib1] Akam M . 1998a. Hox genes in arthropod development and evolution. Biol Bull 195:373–4. 9924778 10.2307/1543151

[bib2] Akam M . 1998b. *Hox* genes, homeosis and the evolution of segment identity: no need for hopeless monsters. Int J Dev Biol 42:445–51. 9654030

[bib3] Aria C , Caron JB. 2015. Cephalic and limb anatomy of a new isoxyid from the Burgess Shale and the role of “stem bivalved arthropods” in the disparity of the frontalmost appendage. PLoS One 10:e0124979. 26038846 10.1371/journal.pone.0124979 PMC4454494

[bib4] Aria C , Zhao F, Zeng H, Guo J, Zhu M. 2020. Fossils from South China redefine the ancestral euarthropod body plan. BMC Evol Biol 20:4. 31914921 10.1186/s12862-019-1560-7 PMC6950928

[bib5] Auman T , Chipman AD. 2017. The evolution of gene regulatory networks that define arthropod body plans. Integr Comp Biol 57:523–32. 28957519 10.1093/icb/icx035

[bib6] Auman T , Vreede BMI, Weiss A, Hester SD, Williams TA, Nagy LM, Chipman AD. 2017. Dynamics of growth zone patterning in the milkweed bug *Oncopeltus fasciatus*. Development 144:1896–905. 28432218 10.1242/dev.142091 PMC5450833

[bib7] Barnett AA , Thomas RH. 2018. Early segmentation in the mite *Archegozetes longisetosus* reveals conserved and derived aspects of chelicerate development. Dev Genes Evol 228:213–7. 29987414 10.1007/s00427-018-0615-x

[bib8] Boxshall GA . 2004. The evolution of arthropod limbs. Biol Rev 79:253–300. 15191225 10.1017/s1464793103006274

[bib9] Boxshall GA . 2013. Arthropod limbs and their development. In: Minelli A, Boxshall G, Fusco G, editors. Arthropod Biology and Evolution. Heidleberg: Springer. p. 241–68.

[bib10] Brown SJ , Patel NH, Denell RE. 1994. Embryonic expression of the single *Tribolium engrailed* homolog. Dev Genet 15:7–18. 8187351 10.1002/dvg.1020150103

[bib11] Browne WE , Price AL, Gerberding M, Patel NH. 2005. Stages of embryonic development in the amphipod crustacean, *Parhyale hawaiensis*. Genesis 42:124–49. 15986449 10.1002/gene.20145

[bib12] Budd GE . 2002. A palaeontological solution to the arthropod head problem. Nature 417:271–5. 12015599 10.1038/417271a

[bib13] Budd GE . 2021. The origin and evolution of the euarthropod labrum. Arthropod Struct Dev 62:101048. 10.1016/j.asd.2021.101048 33862532

[bib14] Campbell LI , Rota-Stabelli O, Edgecombe GD, Marchioro T, Longhorn SJ, Telford MJ, Philippe H, Rebecchi L, Peterson KJ, Pisani D. 2011. MicroRNAs and phylogenomics resolve the relationships of Tardigrada and suggest that velvet worms are the sister group of Arthropoda. Proc Natl Acad Sci 108:15920–4. 21896763 10.1073/pnas.1105499108 PMC3179045

[bib15] Chen JY , Waloszek D, Maas A. 2004. A new ‘great-appendage’ arthropod from the Lower Cambrian of China and homology of chelicerate chelicerae and raptorial antero-ventral appendages. Lethaia 37:3–20.

[bib16] Chipman AD . 2015. An embryological perspective on the early arthropod fossil record. BMC Evol Biol 15:285. 26678148 10.1186/s12862-015-0566-z PMC4683962

[bib17] Chipman AD . 2020. The evolution of the gene regulatory networks patterning the *Drosophila* blastoderm. Curr Top Dev Biol 139:297–324. 32450964 10.1016/bs.ctdb.2020.02.004

[bib18] Chipman AD , Edgecombe GD. 2019. Developing an integrated understanding of the evolution of arthropod segmentation using fossils and evo-devo. Proc Roy Soc B 286:20191881. 10.1098/rspb.2019.1881 PMC6790758 31575373

[bib19] Choe CP , Brown SJ. 2007. Evolutionary flexibility of pair-rule patterning revealed by functional analysis of secondary pair-rule genes, *paired* and *sloppy-paired* in the short-germ insect, *Tribolium castaneum*. Dev Biol 302:281–94. 17054935 10.1016/j.ydbio.2006.09.037 PMC1800430

[bib20] Clark E , Peel AD, Akam M. 2019. Arthropod segmentation. Development 146:170480. 10.1242/dev.170480 31554626

[bib21] Cong P , Ma X, Hou X, Edgecombe GD, Strausfeld NJ. 2014. Brain structure resolves the segmental affinity of anomalocaridid appendages. Nature 513:538–42. 25043032 10.1038/nature13486

[bib22] Damen WGM . 2002. Parasegmental organization of the spider embryo implies that the parasegment is an evolutionary conserved entity in arthropod embryogenesis. Development 129:1239–50. 11874919 10.1242/dev.129.5.1239

[bib23] Damen WGM , Hausdorf M, Seyfarth EA, Tautz D. 1998. A conserved mode of head segmentation in arthropods revealed by the expression pattern of *Hox* genes in a spider. Proc Natl Acad Sci 95:10665–70. 9724761 10.1073/pnas.95.18.10665 PMC27952

[bib24] DiFrisco J , Wagner GP. 2022. Body plan identity: a mechanistic model. Evol Bio :1–19.

[bib25] Dzik J . 2011. The xenusian-to-anomalocaridid transition within the lobopodians. B Soc Paleontol Ital 50:65–74.

[bib26] Edgecombe GD . 2020. Arthropod origins: integrating paleontological and molecular evidence. Annu Rev Ecol Evol Syst 51:1–25.

[bib27] Edgecombe GD , Legg DA. 2014. Origins and early evolution of arthropods. Palaeontology 57:457–68.

[bib28] Farzana L , Brown SJ. 2008. Hedgehog signaling pathway function conserved in *Tribolium* segmentation. Dev Genes Evol 218:181–92. 18392879 10.1007/s00427-008-0207-2 PMC2292471

[bib29] Friedrich M . 2003. Evolution of insect eye development: first insights from fruit fly, grasshopper and flour beetle. Integr Comp Biol 43:508–21. 21680459 10.1093/icb/43.4.508

[bib30] Gallitano-Mendel A , Finkelstein R. 1997. Novel segment polarity gene interactions during embryonic head development in *Drosophila*. Dev Biol 192:599–613. 9441692 10.1006/dbio.1997.8753

[bib31] Giribet G , Edgecombe GD. 2019. The phylogeny and evolutionary history of arthropods. Curr Biol 29:R592–602. 31211983 10.1016/j.cub.2019.04.057

[bib32] Gross V , Epple L, Mayer G. 2021. Organization of the central nervous system and innervation of cephalic sensory structures in the water bear *Echiniscus testudo* (Tardigrada: Heterotardigrada) revisited. J Morphol 282:1298–312. 34129245 10.1002/jmor.21386

[bib33] Haug JT , Waloszek D, Maas A, Liu YU, Haug C. 2012. Functional morphology, ontogeny and evolution of mantis shrimp-like predators in the Cambrian. Palaeontology 55:369–99.

[bib34] Hemmi N , Akiyama-Oda Y, Fujimoto K, Oda H. 2018. A quantitative study of the diversity of stripe-forming processes in an arthropod cell-based field undergoing axis formation and growth. Dev Biol 437:84–104. 29551694 10.1016/j.ydbio.2018.03.001

[bib35] Hombria JC , Garcia-Ferres M, Sanchez-Higueras C. 2021. Anterior Hox genes and the process of cephalization. Front Cell Dev Biol 9:718175. 34422836 10.3389/fcell.2021.718175 PMC8374599

[bib36] Hughes CL , Kaufman TC. 2002. Hox genes and the evolution of the arthropod body plan. Evol Dev 4:459–99. 12492146 10.1046/j.1525-142x.2002.02034.x

[bib37] Hunnekuhl VS , Akam M. 2017. Formation and subdivision of the head field in the centipede *Strigamia maritima*, as revealed by the expression of head gap gene orthologues and *hedgehog* dynamics. EvoDevo 8:18. 29075435 10.1186/s13227-017-0082-x PMC5654096

[bib38] Janssen R . 2012. Segment polarity gene expression in a myriapod reveals conserved and diverged aspects of early head patterning in arthropods. Dev Genes Evol 222:299–309. 22903234 10.1007/s00427-012-0413-9

[bib39] Janssen R , Budd GE, Damen WGM. 2011. Gene expression suggests conserved mechanisms patterning the heads of insects and myriapods. Dev Biol 357:64–72. 21658375 10.1016/j.ydbio.2011.05.670

[bib40] Janssen R , Prpic N-M, Damen WGM. 2004. Gene expression suggests decoupled dorsal and ventral segmentation in the millipede *Glomeris marginata* (Myriapoda: Diplopoda). Dev Biol 268:89–104. 15031107 10.1016/j.ydbio.2003.12.021

[bib41] Jockusch EL . 2017. Developmental and evolutionary perspectives on the origin and diversification of arthropod appendages. Integr Comp Biol 57:533–45. 28957524 10.1093/icb/icx063

[bib42] Kanayama M , Akiyama-Oda Y, Nishimura O, Tarui H, Agata K, Oda H. 2011. Travelling and splitting of a wave of *hedgehog* expression involved in spider-head segmentation. Nat Commun 2:500. 21988916 10.1038/ncomms1510 PMC3207210

[bib43] Kimm MA , Prpic NM. 2006. Formation of the arthropod labrum by fusion of paired and rotated limb-bud-like primordia. Zoomorphology 125:147–55.

[bib44] Lan T , Zhao Y, Zhao F, He Y, Martinez P, Strausfeld NJ. 2021. Leanchoiliidae reveals the ancestral organization of the stem euarthropod brain. Curr Biol 31:1–8. 34416180 10.1016/j.cub.2021.07.048

[bib45] Legg DA , Sutton MD, Edgecombe GD. 2013. Arthropod fossil data increase congruence of morphological and molecular phylogenies. Nat Commun 4:2485. 24077329 10.1038/ncomms3485

[bib46] Lev O , Chipman AD. 2021. Development of the pre-gnathal segments in the milkweed bug *Oncopeltus fasciatus* suggests they are not serial homologs of trunk segments. Front Cell Dev Biol 9:695135. 34422818 10.3389/fcell.2021.695135 PMC8378449

[bib47] Loesel R , Wolf H, Kenning M, Harzsch S, Sombke A. 2013. Architectural principles and evolution of the arthropod central nervous system. In: Minelli A, Boxshall G, Fusco G, editors. Arthropod Biology and Evolution. Heidleberg: Springer. p. 241–68.

[bib48] Ma X , Hou X, Edgecombe GD, Strausfeld NJ. 2012. Complex brain and optic lobes in an early Cambrian arthropod. Nature 490:258–61. 23060195 10.1038/nature11495

[bib49] Martin C , Jahn H, Klein M, Hammel JU, Stevenson PA, Homberg U, Mayer G. 2022. The velvet worm brain unveils homologies and evolutionary novelties across panarthropods. BMC Biol 20:26. 35073910 10.1186/s12915-021-01196-w PMC9136957

[bib50] Mayer G , Whitington PM, Sunnucks P, Pfluger HJ. 2010. A revision of brain composition in Onychophora (velvet worms) suggests that the tritocerebrum evolved in arthropods. BMC Evol Biol 10:1–9. 10.1186/1471-2148-10-255 PMC2933641 20727203

[bib51] Mittmann B , Scholtz G. 2003. Development of the nervous system in the “head” of *Limulus polyphemus* (Chelicerata: Xiphosura): morphological evidence for a correspondence between the segments of the chelicerae and of the (first) antennae of Mandibulata. Dev Genes Evol 213:9–17. 12590348 10.1007/s00427-002-0285-5

[bib52] Miyawaki K , Mito T, Sarashina I, Zhang HJ, Shinmyo Y, Ohuchi H, Noji S. 2004. Involvement of *Wingless/Armadillo* signaling in the posterior sequential segmentation in the cricket, *Gryllus bimaculatus* (Orthoptera), as revealed by RNAi analysis. Mech Dev 121:119–30. 15037314 10.1016/j.mod.2004.01.002

[bib53] Ortega-Hernández J . 2016. Making sense of ‘lower’ and ‘upper’ stem-group Euarthropoda, with comments on the strict use of the name Arthropoda von Siebold, 1848. Biol Rev Camb Philos Soc 91:255–73. 25528950 10.1111/brv.12168

[bib54] Ortega-Hernández J , Janssen R, Budd GE. 2017. Origin and evolution of the panarthropod head—A palaeobiological and developmental perspective. Arthropod Struct Dev 46:354–79. 27989966 10.1016/j.asd.2016.10.011

[bib55] Park TS , Kihm JH, Woo J, Park C, Lee WY, Smith MP, Harper DAT, Young F, Nielsen AT, Vinther J. 2018. Brain and eyes of *Kerygmachela* reveal protocerebral ancestry of the panarthropod head. Nat Commun 9:1019. 29523785 10.1038/s41467-018-03464-w PMC5844904

[bib56] Peel AD , Chipman AD, Akam M. 2005. Arthropod segmentation: beyond the *Drosophila* paradigm. Nat Rev Genet 6:905–16. 16341071 10.1038/nrg1724

[bib57] Posnien N , Bashasab F, Bucher G. 2009. The insect upper lip (labrum) is a nonsegmental appendage-like structure. Evol Dev 11:480–8. 19754705 10.1111/j.1525-142X.2009.00356.x

[bib58] Posnien N , Bucher G. 2010. Formation of the insect head involves lateral contribution of the intercalary segment, which depends on *Tc-labial* function. Dev Biol 338:107–16. 19913530 10.1016/j.ydbio.2009.11.010

[bib59] Posnien N , Schinko JB, Kittelmann S, Bucher G. 2010. Genetics, development and composition of the insect head—A beetle's view. Arthropod Struct Dev 39:399–410. 20800703 10.1016/j.asd.2010.08.002

[bib60] Richter S , Loesel R, Purschke G, Schmidt-Rhaesa A, Scholtz G, Stach T, Vogt L, Wanninger A, Brenneis G, Döring C et al. 2010. Invertebrate neurophylogeny: suggested terms and definitions for a neuroanatomical glossary. Front Zool 7:29. 21062451 10.1186/1742-9994-7-29 PMC2996375

[bib61] Rogers BT , Peterson MD, Kaufman TC. 2002. The development and evolution of insect mouthparts as revealed by the expression patterns of gnathocephalic genes. Evol Dev 4:96–110. 12004967 10.1046/j.1525-142x.2002.01065.x

[bib62] Scholtz G . 2015. Heads and brains in arthropods: 40 years after the “endless dispute.” In: Schmidt-Rhaesa A, Harzsch S, Purschke G, editors. Structure and Evolution of Invertebrate Nervous Systems. Oxford: Oxford University Press. p. 402–10.

[bib63] Schönauer A , Paese CL, Hilbrant M, Leite DJ, Schwager EE, Feitosa NM, Eibner C, Damen WG, McGregor AP. 2016. The Wnt and Delta-Notch signalling pathways interact to direct pair-rule gene expression via *caudal* during segment addition in the spider *Parasteatoda tepidariorum*. Development 143:2455–63. 27287802 10.1242/dev.131656

[bib64] Smith FW , Bartels PJ, Goldstein B. 2017. A hypothesis for the composition of the tardigrade brain and its implications for panarthropod brain evolution. Integr Comp Biol 57:546–59. 28957526 10.1093/icb/icx081

[bib65] Stahi R , Chipman AD. 2016. Blastoderm segmentation in *Oncopeltus fasciatus* and the evolution of arthropod segmentation mechanisms. Proc R Soc Lond B 283:20161745. 10.1098/rspb.2016.1745 PMC5069518 27708151

[bib66] Steinmetz PR , Urbach R, Posnien N, Eriksson J, Kostyuchenko RP, Brena C, Guy K, Akam M, Bucher G, Arendt D. 2010. *Six3* demarcates the anterior-most developing brain region in bilaterian animals. Evodevo 1:14. 21190549 10.1186/2041-9139-1-14 PMC3025827

[bib67] Strausfeld NJ , Ma X, Edgecombe GD, Fortey RA, Land MF, Liu Y, Cong P, Hou X. 2016. Arthropod eyes: the early Cambrian fossil record and divergent evolution of visual systems. Arthropod Struct Dev 45:152–72. 26276096 10.1016/j.asd.2015.07.005

[bib68] Telford MJ , Thomas RH. 1998. Expression of homeobox genes shows chelicerate arthropods retain their deutocerebral segment. Proc Natl Acad Sci 95:10671–5. 9724762 10.1073/pnas.95.18.10671 PMC27953

[bib69] Tomoyasu Y , Ohde T, Clark-Hachtel C. 2017. What serial homologs can tell us about the origin of insect wings. F1000Res 6:268. 28357056 10.12688/f1000research.10285.1 PMC5357031

[bib70] Urbach R , Technau GM. 2003. Segment polarity and DV patterning gene expression reveals segmental organization of the *Drosophila* brain. Development 130:3607–20. 12835379 10.1242/dev.00532

[bib71] von Dassow G , Meir E, Munro EM, Odell GM. 2000. The segment polarity network is a robust developmental module. Nature 406:188–92. 10910359 10.1038/35018085

[bib72] Wagner GP . 2007. The developmental genetics of homology. Nat Rev Genet 8:473–9. 17486120 10.1038/nrg2099

[bib73] Wagner GP . 2014. Homology, Genes and Evolutionary Innovation. Princeton and Oxford: Princeton University Press.

[bib74] Wagner GP , Gauthier JA. 1999. 1,2,3 = 2,3,4: a solution to the problem of the homology of the digits in the avian hand. Proc Natl Acad Sci 96:5111–6. 10220427 10.1073/pnas.96.9.5111 PMC21825

[bib75] Zeng H , Zhao F, Niu K, Zhu M, Huang D. 2020. An early Cambrian euarthropod with radiodont-like raptorial appendages. Nature 588:101–5. 33149303 10.1038/s41586-020-2883-7

